# Supplementary material for: Comprehensive Characterization of the Odor-Active Compounds in Different Processed Varieties of Yunnan White Tea (Camellia sinensis) by GC×GC-O-MS and Chemometrics
Source: Foods. 2025 Jan 15;14(2):271. doi: 10.3390/foods14020271 (PMC11764680; doi:10.3390/foods14020271)
Supplement: Supplementary file 1 [file foods-14-00271-s001.zip › Supplementary Figures.pdf]

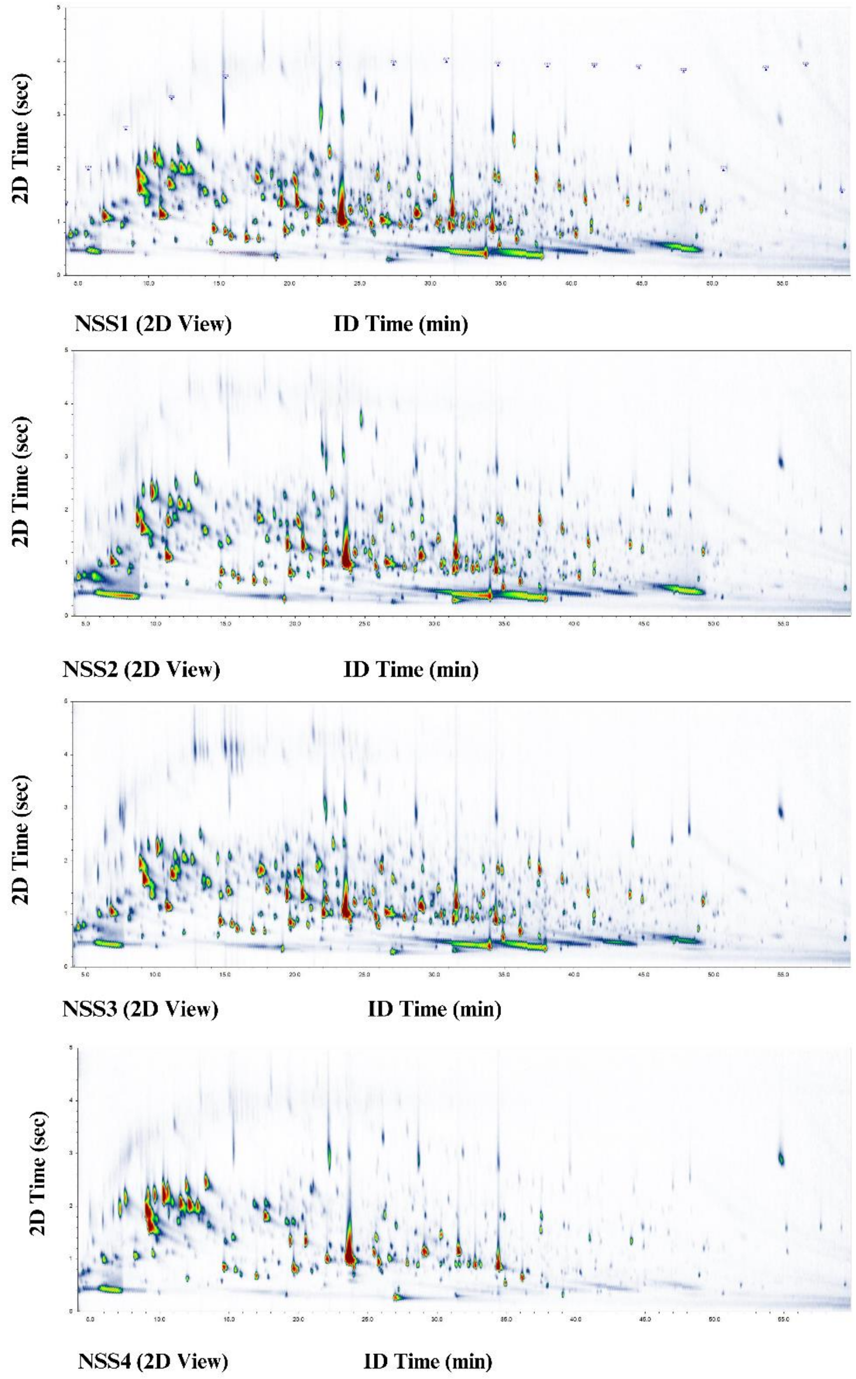

Figure S1a. 3D peaks for unshaken white tea varieties (NSS1, NSS2, NSS3, and NSS4).

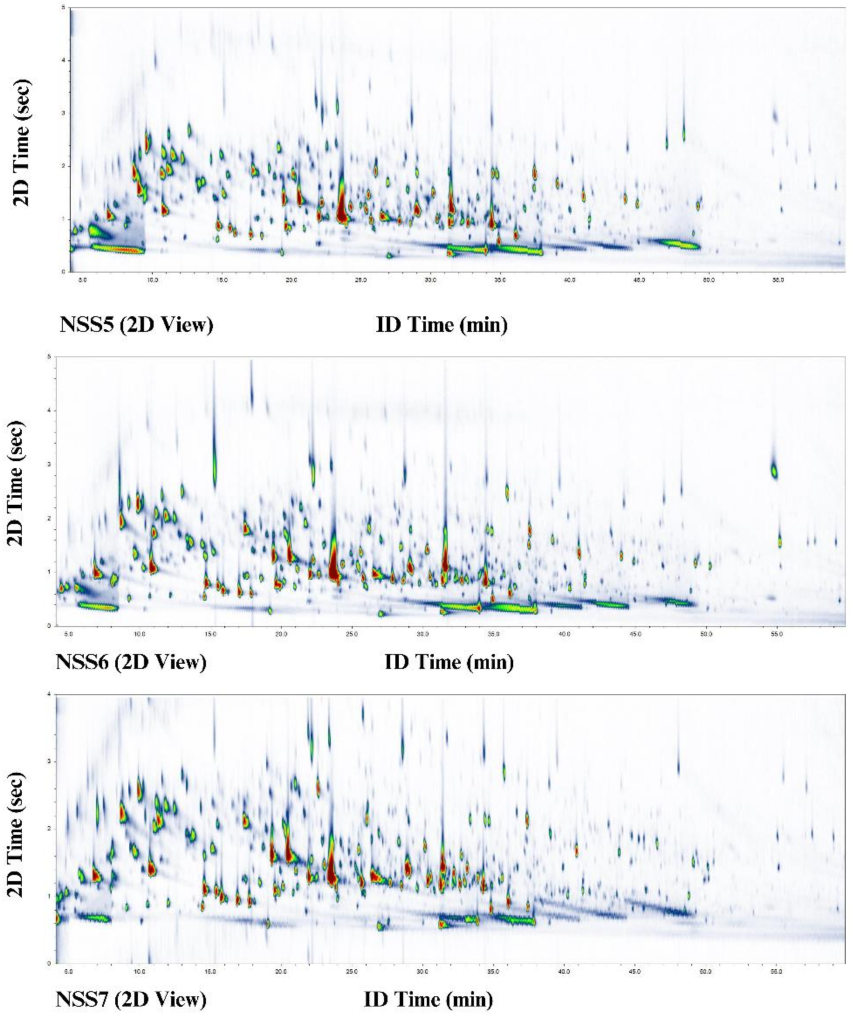

Figure S1b. 3D peaks for unshaken white tea varieties (NSS5, NSS6, and NSS7).

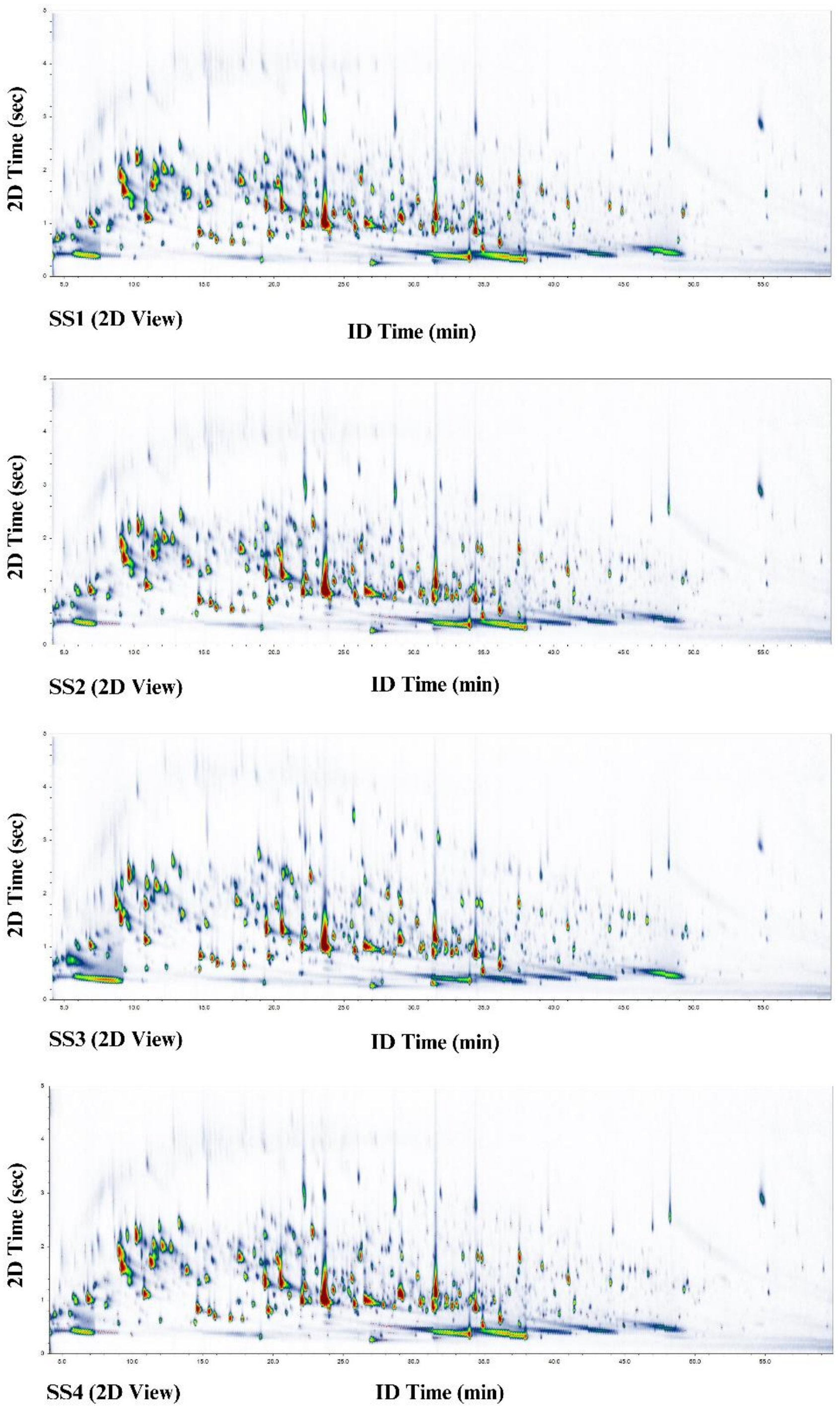

Figure S1c. 3D peaks for shaken white tea varieties (SS1, SS2, SS3, and SS4).

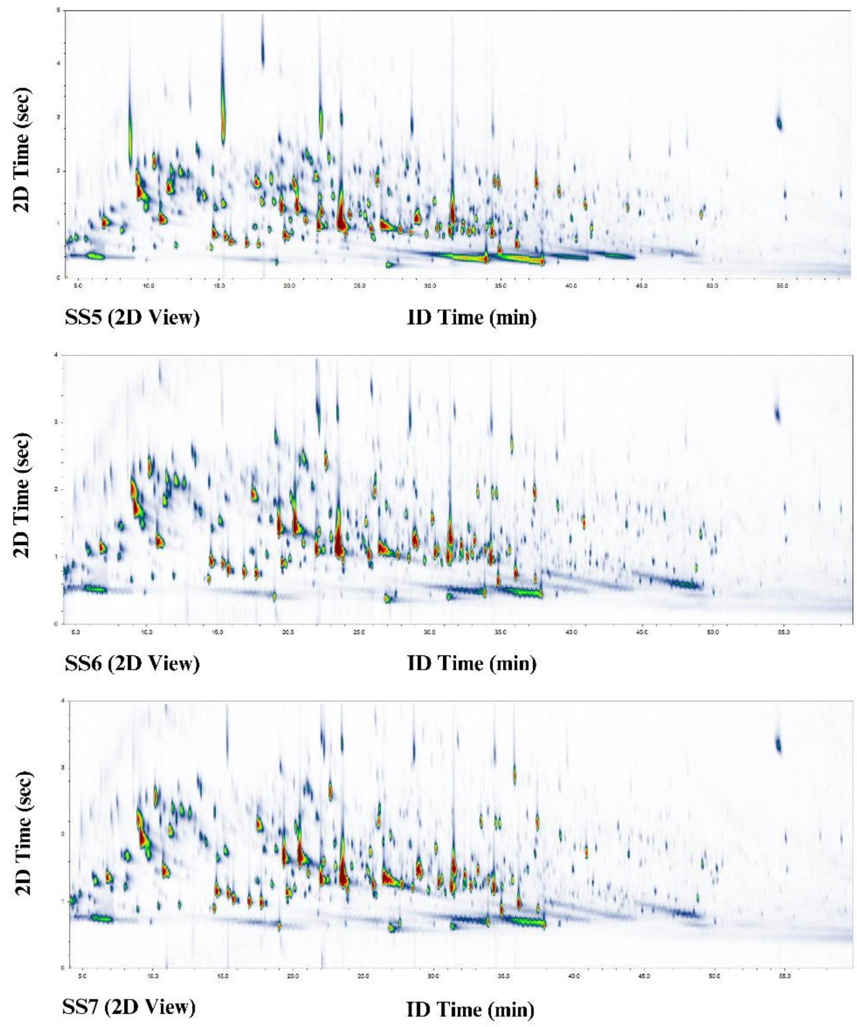

Figure S1d. 3D peaks for shaken white tea varieties (SS5, SS6, and SS7).

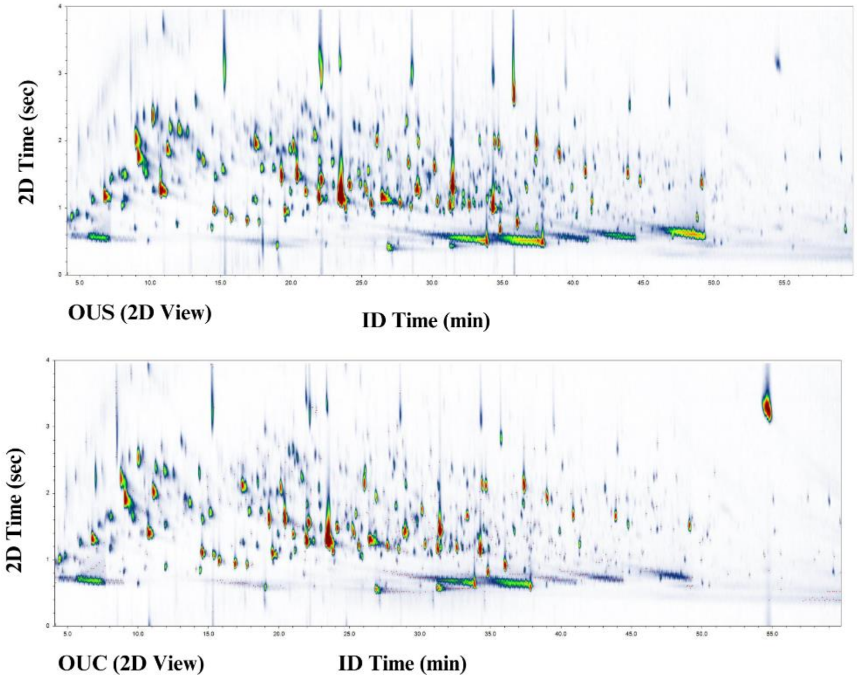

Figure S1e. 3D peaks for processed white tea varieties (OUS and OUC).

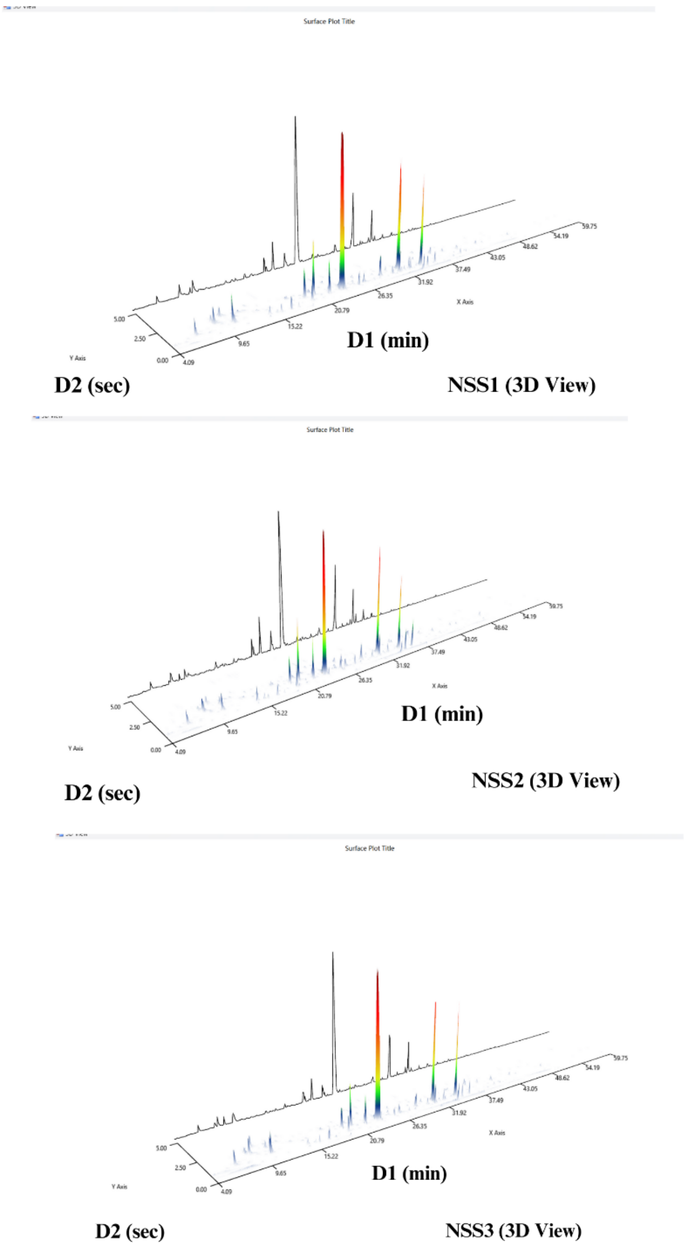

Figure S2a. 2D peaks for unshaken white tea varieties (NSS1, NSS2, and NSS3).

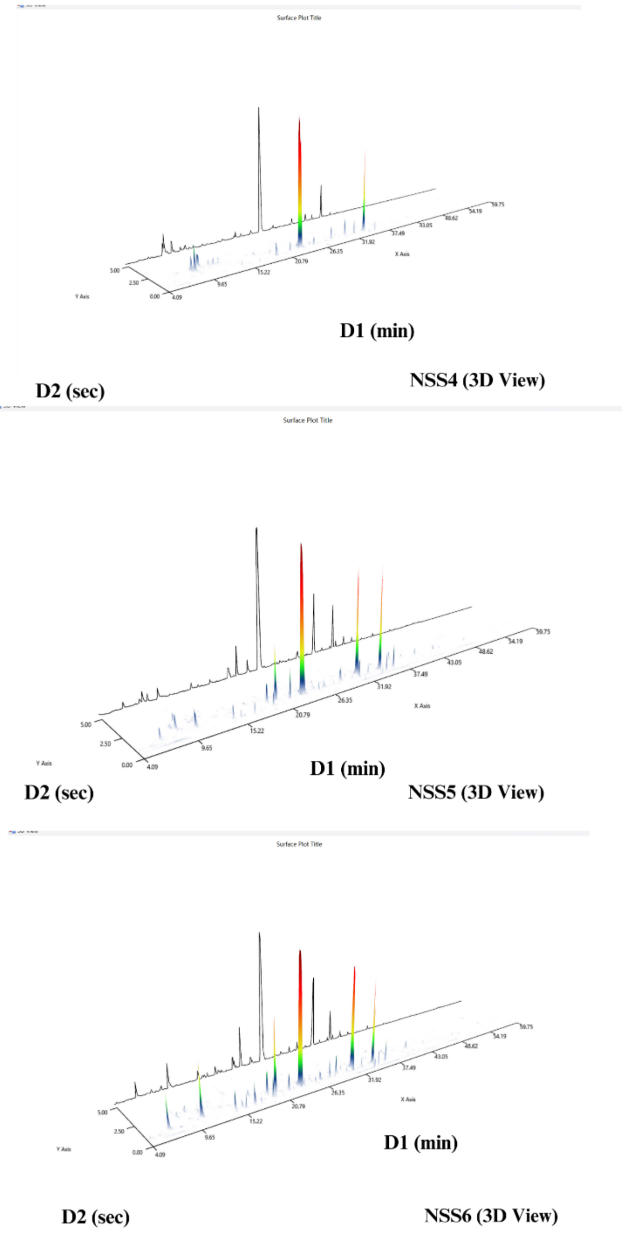

Figure S2b. 2D peaks for unshaken white tea varieties (NSS4, NSS5, and NSS6).

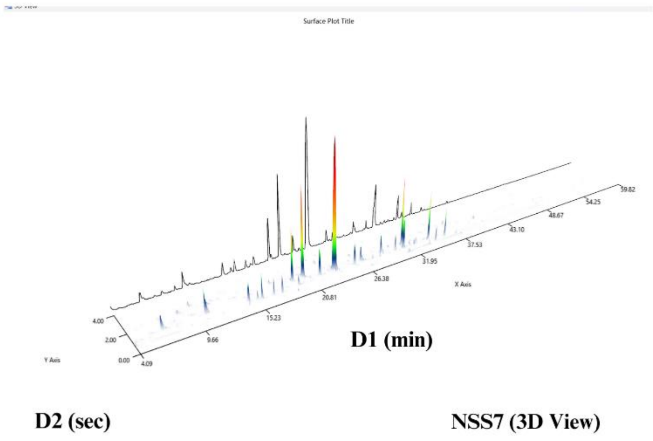

Figure S2c. 2D peaks for the unshaken white tea variety NSS7.

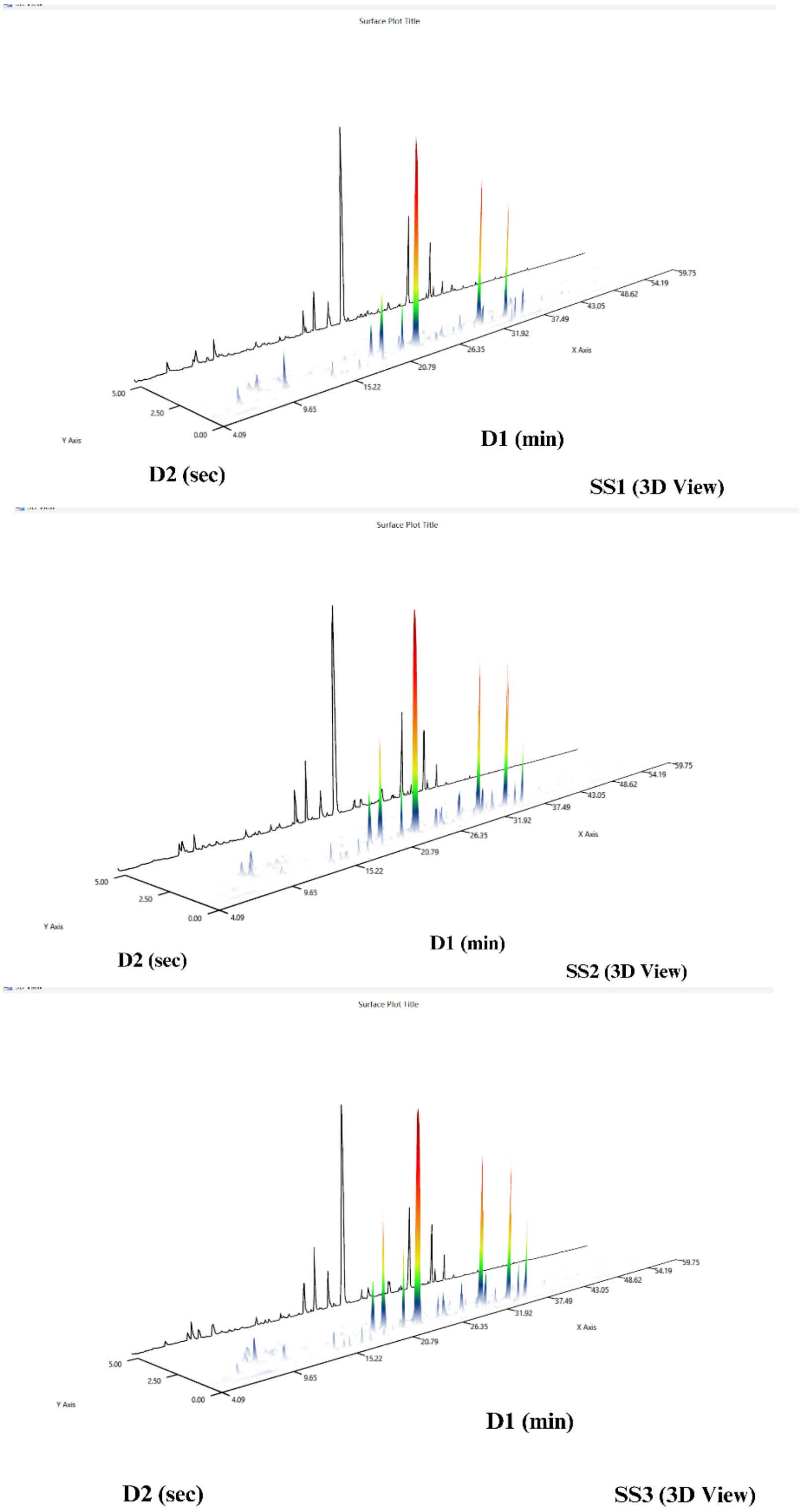

Figure S2d. 2D peaks for shaken white tea varieties (SS1, SS2, and SS3).

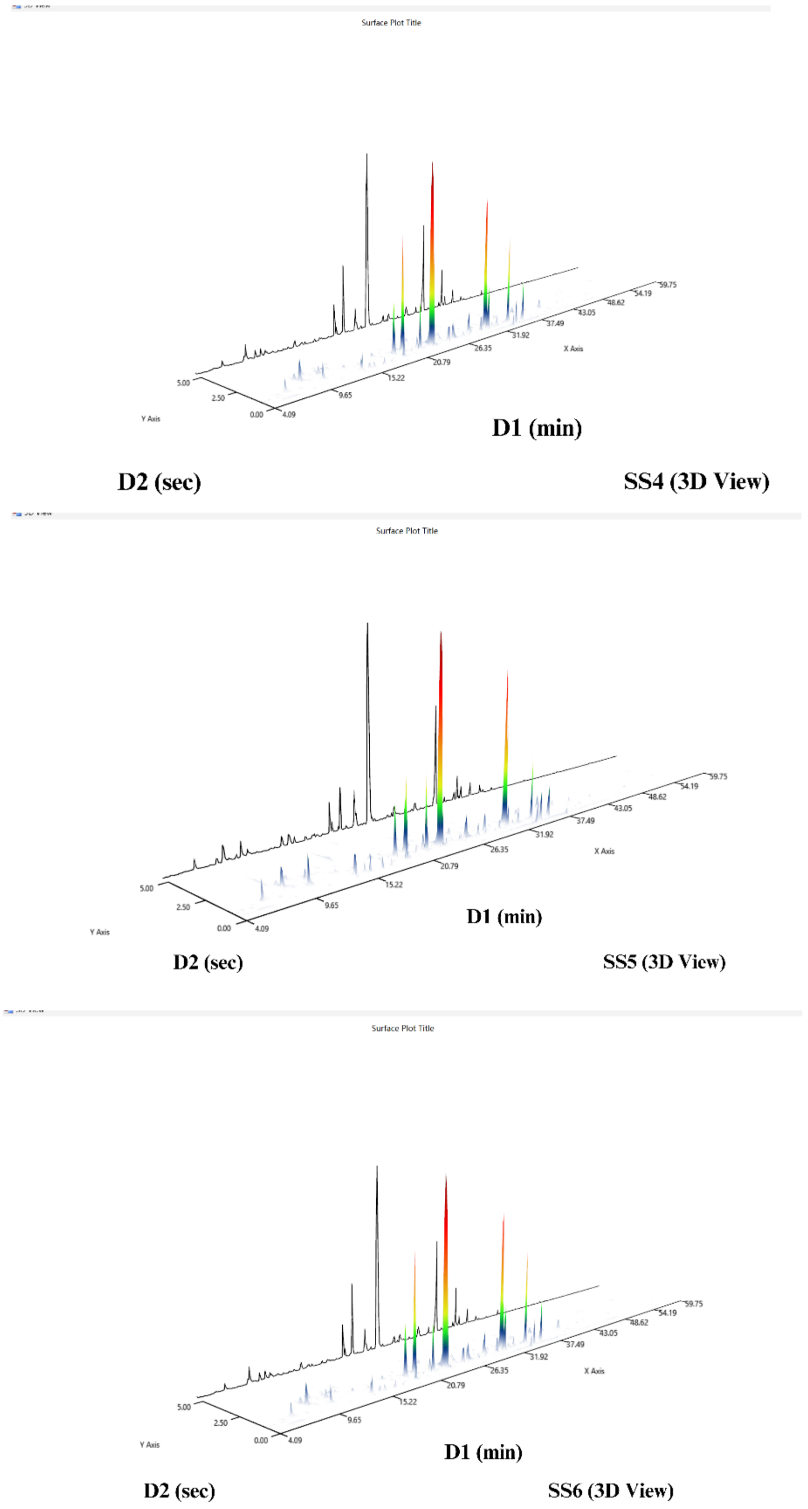

Figure S2e. 2D peaks for shaken white tea varieties (SS4, SS5, and SS6).

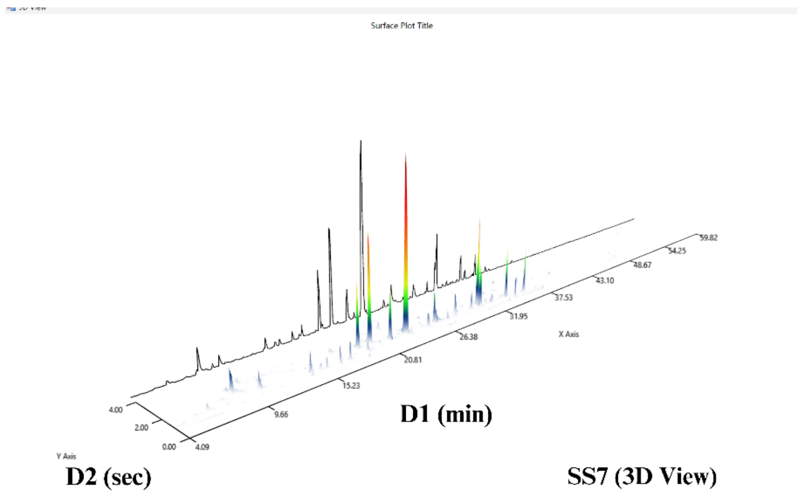

Figure S2f. 2D peaks for the shaken white tea variety SS7.

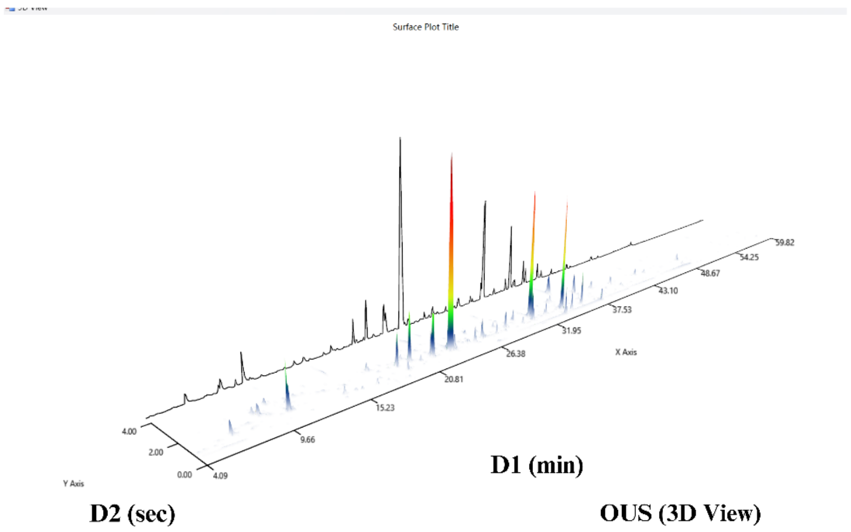

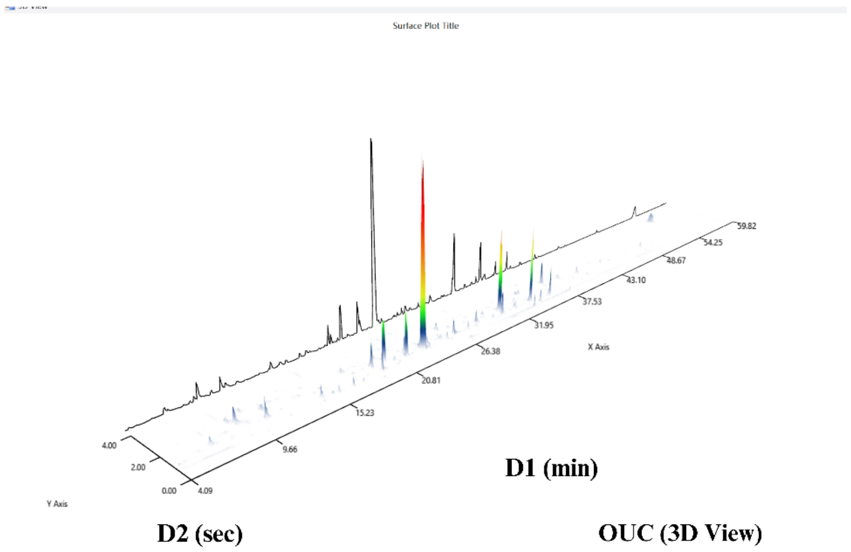

Figure S2g. 2D peaks for processed white tea varieties (OUS and OUC).

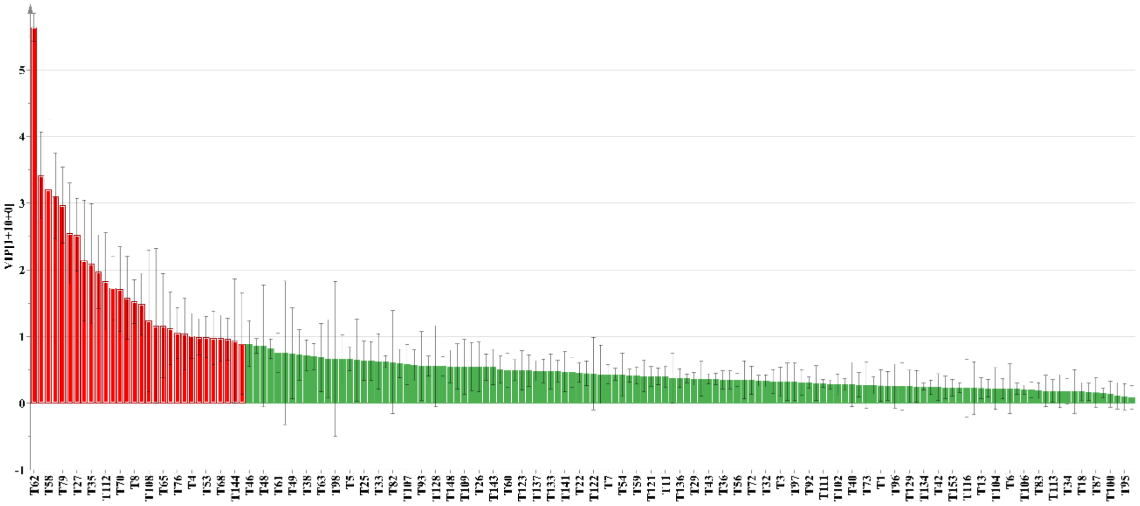

Figure S3. VIP plots of different white tea varieties analyzed through OPSD-DA.
