# Supplementary material for: Comprehensive Characterization of the Odor-Active Compounds in Different Processed Varieties of Yunnan White Tea (Camellia sinensis) by GC×GC-O-MS and Chemometrics
Source: Foods. 2025 Jan 15;14(2):271. doi: 10.3390/foods14020271 (PMC11764680; doi:10.3390/foods14020271)
Supplement: Supplementary file 1 [file foods-14-00271-s001.zip › Supplementary Table S1.pdf]

Table S1. Relative concentrations of volatile compounds in the 16 white tea varieties.

Volatile compounds	SS1	SS2	SS3	SS4	SS5	SS6	SS7	NSS1	NSS2	NSS3	NSS4	NSS5	NSS6	NSS7	OUS	OUC
	Conc. (μg/kg)															
**Nitrogen-containing**																
1-ethyl pyrrole	20.6±1.37	0	0	0	0	0	0	0	0	0	59.2±4.42	0	0	0	0	0
**Oxygen heterocyclic compounds**																
2-ethyl-5-methyl furan	0	77.72±4.77	0	38.81±1.22	17.98±2.71	0	0	0	0	14.56±0.92	0	0	0	18.63±3.29	0	0
2-pentyl furan	579.97±38.64	248.9±1.84	632.61±12.22	712.53±22.44	571.37±43.43	197.13±1.73	210.91±18.16	553.46±9.55	622.49±34.43	872.75±20.48	45.97±2.03	328.94±20.05	167.66±5.39	77.24±9.65	705.81±22.05	2500.78±78.12
(E)-2-(2-pentenyl) furan	295.62±19.69	94.7±0.7	212.36±4.1	263.15±8.29	134.49±10.22	63.41±0.56	52.05±4.48	178.53±3.08	281.5±15.57	232.33±5.45	0	147.9±9.02	87±2.8	10.71±1.61	0	635.25±19.84
**Aldehydes**																
3-methylbutanal	131.2±5.27	44.21±2.71	63.25±1.05	29.77±0.94	30.69±4.63	34.4±0.62	67.34±5.8	11.55±16.3	107.12±5.93	30.6±1.93	0	25.15±0.85	0	12.07±2.13	29.08±0.64	81.64±1.8
Pentanal	0	45.36±2.78	50.32±0.83	52.75±1.66	71.79±10.84	37.95±0.68	44.94±3.87	96.23±9.85	0	105.42±6.66	26.81±0.91	82.62±2.79	34.75±1.2	34.38±6.06	109.68±2.42	292.79±6.47
Hexanal	31.73±1.27	247.9±15.21	622.14±10.28	718.28±22.62	788.8±119.1	496.96±8.91	376.04±25.16	1331.91±136.33	1241.96±68.7	960.86±76.22	21.22±0.72	1089±36.71	10.91±3.23	297.4±44.62	1741.54±38.48	2612.57±57.73
(E)-2-methyl-2-butenal	0	0	0	0	0	0	29.97±2.58	0	0	19.53±1.23	0	0	0	0	0	0
2-methyl-2-Butenal	25.03±1	0	23.58±0.39	15.67±0.49	0	22.69±0.41	0	0	0	0	0	15.97±0.54	71.38±2.47	0	0	0
(Z)-3-Hexenal	0	0	0	0	0	0	0	0	74.24±4.11	0	0	81.18±2.74	0	0	89.65±1.98	0
(E)-2-pentenal	88.57±3.56	11.15±0.68	0	167.92±5.29	71.6±10.81	57.75±1.04	55.49±4.78	108.15±11.07	117.41±6.49	0	0	91.59±3.09	74.51±2.58	38.27±5.74	125.58±2.77	237.55±7.42
2-methyl-2-pentenal	0	28.43±1.74	0	0	0	0	0	0	0	15.82±1	0	32.68±1.1	0	0	0	0
Heptanal	0	78.46±0.58	0	0	0	97.35±0.86	79.95±6.88	152.01±2.62	121.51±11.43	0	0	138.33±8.43	69.18±2.22	46.73±7.01	208.83±6.52	0
5-methyl hexanal	177.6±11.83	0	145.03±2.8	0	0	0	0	0	0	0	25.98±1.15	0	0	0	0	547.94±17.12
(E)-2-hexenal	2079.54±138.54	0	0	957.48±30.15	1334.29±101.43	0	0	0	72.21±14.72	49.2±1.15	0	0	0	0	0	4049.04±126.49
2-Hexenal	0	421.93±3.12	1029.18±19.89	0	0	1232.66±10.84	814.26±70.11	2036.6±35.15	1969.54±108.94	1195.53±28.05	0	1646.12±100.36	980.31±1337.86	698.22±104.75	0	0
(Z)-4-heptenal	28.73±1.91	0	0	0	11.63±0.88	0	10.04±0.86	0	16.99±0.94	18.94±0.44	0	0	0	0	0	0
Octanal	105.11±7	0	32.05±0.62	47.12±1.48	60.34±4.59	44.95±0.4	47.52±4.09	65.79±1.14	0	87.72±2.06	54.23±2.4	0	39.33±1.26	0	0	240.54±7.51

Nonanal	492.39±32.8	273.18±2.02	396.05±7.65	432.3±13.61	367.5±27.94	266.22±2.34	261.39±22.5	370.53±6.39	377.32±26.84	368.38±8.64	223.97±9.9	291.23±17.75	378.01±12.16	208.27±31.24	1035.9±32.36	1601.11±50.02
(E,E)-2,4-hexadienal	0	0	47.5±0.92	47.82±1.51	63.75±4.85	48.96±0.43	0	75.51±1.3	0	36.1±36.18	0	48.13±2.93	75.07±2.41	12.17±11.1	185.3±5.79	206.25±6.44
(E)-2-octenal	0	20.76±0.15	49.47±0.96	41.83±1.32	79.25±6.02	48.85±0.43	55.14±4.75	39.35±0.68	28.98±19.91	132.14±111.05	0	18.77±1.14	22.06±0.71	17.51±2.63	110.12±3.44	232.04±7.25
Furfural	0	0	12.94±0.25	0	0	0	24.15±2.08	0	0	16.69±0.39	0	0	0	0	0	0
(E,E)-2,4-heptadienal	90.93±6.06	110.53±0.82	181.26±3.5	151.33±4.76	321.15±24.41	0	25.86±2.23	17.62±0.3	45.64±50.42	70.69±75.01	0	37.24±2.27	67.31±55.35	55.43±8.32	532.39±16.63	942.79±29.45
Decanal	83.06±5.53	50.89±0.38	0	115.37±3.63	179.72±13.66	64.95±0.57	50.01±4.31	81.14±1.4	0	158.61±3.72	9.66±0.43	36.82±2.24	78.38±2.52	21.77±3.27	0	425.68±13.3
Benzaldehyde	2598.98±173.15	1774.7±13.11	3532.55±68.26	2575.49±81.09	2612.88±198.62	2020.09±17.77	2137.31±181.37	783.41±1059.76	39.01±2.16	2389.54±22.53	316.88±14	1637.36±99.82	739.2±23.77	1021.98±124.95	4093.11±127.87	275.34±8.6
(E)-2-Nonenal	54.5±3.63	34.17±0.25	68.57±1.32	67.5±2.13	69.98±5.32	0	47.45±4.09	46.32±0.8	0	0	0	16.18±0.99	40.2±1.29	20.33±3.05	0	162.23±5.07
(E,Z)-2,6-nonadienal	24.79±1.65	11.52±0.09	29.5±0.57	37.34±1.18	32.61±2.48	21.07±0.19	15.69±1.35	26.95±0.47	33.73±1.87	28.72±0.67	0	16.95±1.03	30.58±0.98	10.14±1.52	135.5±4.23	146.92±4.59
Beta-cyclocitral	354.79±23.64	101.93±0.75	169.4±3.27	244.98±7.71	0	0	0	0	245.45±13.58	373.32±8.76	45.3±2	261.67±15.95	88.9±2.86	98.63±14.8	0	1685.09±52.64
Safranal	141.45±9.42	23.63±0.17	48.42±0.94	43.5±1.37	47.09±3.58	53.05±0.47	55.91±4.81	83.66±1.44	90.09±4.98	164.26±3.85	25.27±1.12	125.3±7.64	34.61±1.11	23.97±3.6	139.99±4.37	575.44±17.98
(E)-2-decenal	0	0	0	0	0	0	0	0	0	25.74±0.6	12.77±0.56		0	0	0	0
Benzeneacetaldehyde	1196.6±79.72	1189.4±8.79	108.96±2.11	49.46±1.56	1448.25±110.09	1666.34±14.66	2205.63±160.68	23.94±0.41	645.47±473.57	879.7±905.26	62.02±2.74	799.48±48.74	545.85±10	722.41±108.37	2452.67±76.62	4232.14±132.21
2-butyl-2-octenal	68.42±4.56	37.21±0.27	19.7±0.38	29.41±0.93	52.04±3.96	0	0	35.68±2.35	0	29.45±14.1	0	37.44±2.28	15.89±0.51	0	67.54±2.11	193.9±6.06
Neral	74.16±4.94	479.95±3.55	68.78±1.33	63.55±2	43.5±3.31	67.57±0.59	35.48±3.05	88.3±1.52	241.59±185.96	136.57±112.12	11.86±0.52	72.7±4.43	167.94±125.42	79.49±61.2	0	273.29±8.54
(E,E)-2,4-decadienal	0	18.06±0.13	30.77±0.59	0	0	0	0	0	0	0	0	0	0	0	1493.59±46.66	0
**Alcohols**																
1-Penten-3-ol	51.99±2.09	28.84±0.21	46.53±0.9	31.33±0.99	28.59±2.17	25.95±0.23	26.82±2.31	45.04±4.61	149.99±14.11	29.93±1.89	0	0	23.77±0.82	12.97±1.95	33.19±0.73	0
3-methyl-1-butanol	19.22±1.28	0	40.77±0.79	0	0	0	0	0	33.48±1.85	21.04±0.49	0	0	11.18±0.36	0	0	0
2-methyl-1-butanol	0	38.46±0.28	0	13.13±0.41	0	0	31.83±2.74	0	0	0	0	0	0	0	0	0
1-Pentanol	34.41±2.29	24.35±0.18	46.73±0.9	35.48±1.12	41.57±3.16	18.81±0.17	21.02±1.81	42.14±0.73	36.47±2.02	38.63±3.97	25.29±1.12	0	0	0	0	234.1±7.31
2-Heptanol	617.71±41.15	707.54±5.23	776.8±15.01	799.83±25.18	871.77±66.27	798.99±7.03	686.99±59.15	458.57±7.91	396.2±23.24	752.91±11.05	168.18±8.36	801.05±48.84	577.56±15.18	356.79±53.52	626.99±19.59	2275.55±71.09
(Z)-2-penten-1-ol	75.3±5.02	94.52±0.7	109.97±2.12	57.58±1.81	56.2±4.27	110.8±0.97	109.42±9.42	94.97±1.64	79.32±7.59	41.86±0.98	0	34.7±2.12	63.35±2.04	60.53±9.08	178±5.56	267.58±8.36

1-Hexanol	199.99±13.32	425.49±3.14	369.93±7.15	226.87±7.14	410.17±31.18	607.25±5.34	346.56±29.84	372.7±16.99	316.33±17.5	241.34±5.66	19.03±0.84	177.98±10.85	202.04±1.89	295.93±44.39	211.62±6.61	1149.36±35.91
3-Hexen-1-ol	0	0	0	258.7±8.15	334.94±25.46	0	17.55±1.51	0	41.55±46.92	0	82.26±3.64	0		88.76±112.74	0	1429.77±44.67
(Z)-3-hexen-1-ol	390±25.98	481±3.55	524.73±10.14	0	0	519.17±4.57	457.92±39.43	423.66±573.21	318.06±442.44	222.84±300.27	0	487.01±29.69	460.92±14.82	0	338.88±10.59	0
(E)-2-hexen-1-ol	90.87±6.05	0	0	83.17±2.62	163.63±12.44	598.13±5.26	540.28±46.52	161.5±11.42	129.86±7.78	0	10.33±0.46	153.78±9.38	102.19±3.29	254.94±31.78	0	0
(Z)-2-hexen-1-ol	0	383.22±2.83	305.27±5.9	0	0	0	0	0	0	105.55±2.55	0	0	0	0	0	0
2-Octanol	25.91±1.73	31.04±0.23	0	0	54.71±4.16	0	0	28.99±0.5	0	50.52±1.19	11.04±0.49	35.69±2.18	0	0	0	141.87±4.43
(Z)-linalool oxide (furanoid)	2000.68±133.29	7141.3±52.75	0	3469.88±109.25	1900.84±144.5	0	3654.6±314.65	0	2139.33±118.33	2147.28±50.39	164.3±7.26	1989.83±121.31	1259.82±26.94	2371.89±346.05	0	4697.87±146.76
1-Octen-3-ol	524.55±34.95	321.22±2.37	388.35±7.5	737.85±23.23	729.3±55.44	241.34±2.12	348.23±29.98	826.17±18.43	842.43±46.6	853.45±13.22	421.33±19.04	873.71±53.27	473.59±10.33	152.55±22.88	581.43±18.16	2556.11±79.85
1-Heptanol	69.2±4.61	35.32±0.26	38.35±0.74	50.18±1.58	81.04±6.16	75.56±0.66	0	0	45.24±14.17	0	49.2±2.17	62.95±3.84	113.4±3.65	24.61±3.69	79.49±2.48	231.05±7.22
cis-Linaloloxide	44.08±2.94	32.8±0.24	3883.2±75.03	57.82±1.82	0	29.27±0.26	0	13.97±17.03	0	17.18±0.4	0	0	12.51±7.36	0	54.53±1.7	124.64±3.89
6-methyl-5-hepten-2-ol	28.78±1.92	31.81±0.23	0	34.22±1.08	49.19±3.74	59.5±0.52	46.51±4	60.07±1.04	25.77±1.43	52.6±1.23	8.03±0.35	32.54±1.98	30.4±0.98	29.6±4.44	33.69±1.05	87.18±2.72
Nerol oxide	0	24.7±0.18	41.54±0.8	0	0	0	61.32±5.28	37.29±0.64	34.98±1.94	56.86±1.33	3.97±0.18	28.14±1.72		32.12±4.82	0	73.28±2.29
(E)-linalool oxide (furanoid)	4175.68±278.19	50.33±0.37	6319.41±122.11	6999.45±220.39	3458.2±262.88	8147.73±71.66	7386.51±633.71	4085.97±2525.98	3204.86±177.27	4054.02±95.13	271.31±11.99	4319.36±263.33	3325.85±106.96	4683.62±702.62	5085.3±158.86	11337.42±354.17
2-ethyl-1-hexanol	0	11.77±0.09	0	0	0	18.18±0.16	19.63±1.69	22.98±0.4	14.66±0.81	25.96±0.61	0	0	0	0	0	146.56±4.58
2-Nonanol	72.74±4.85	0	0	93.34±2.94	68.74±5.23	0	75±6.46	40.04±0.69	40.76±2.25	129.6±42.78	6.81±2.43	59.33±3.62	79.87±1.37	0	96.04±3	277.62±8.67
Dihydrolinalool	0	0	0	32.37±1.02	0	91.6±0.81	75.57±6.51	86.48±2.91	0	0	12.36±0.55	0	16.33±0.53	141.71±21.26	0	178.78±5.58
Linalool	32457.33±2162.39	16357.13±120.83	42069.08±812.87	36573.89±1151.58	27470.9±2088.25	22486.99±197.77	15164.82±618.58	30044.71±9789.91	37639.72±9722.1	27655.4±270.08	17856.61±3424.26	46267.04±2820.66	13251.09±18526.0	13272.13±1991.0	41456.02±1295.06	98772.19±3085.5
1-Octanol	122.54±8.16	60.05±0.44	78.87±1.52	0	281.02±21.36	0	147.96±12.74	120.71±2.08	0	275.5±6.46	51.67±2.28	94.14±5.74	177.83±5.72	0	0	939.13±29.34
4-terpinenol	125.83±8.38	0	135.86±2.63	220.99±6.96	0	0	92.23±7.94	0	0	194.36±4.56	0	231.64±14.12	88.45±2.84	0	0	350.59±10.95
1,5,7-Octatrien-3-ol, 3,7-dimethyl-	545.71±36.36	783.07±5.78	862.21±16.66	915.85±28.84	276.46±21.02	0	240.65±332.97	0	0	862.65±20.24	0	382.49±23.32	216.32±6.96	302.91±419.96	0	1032.35±32.25
1-Nonanol	401.18±26.73	314.47±2.32	210.19±4.06	246.83±7.77	374.82±28.49	266.39±2.34	228.28±19.65	146.5±2.53	184.95±10.23	435.73±10.22	67.17±2.97	278.4±16.97	514.53±7.97	162.15±104.65	862.44±26.94	1317.87±41.17
α-Terpineol	0	0	0	0	997.09±75.8	0	0	0	1489.03±92.96	475.59±653.63	0	840.25±51.23	0	0	0	2619.64±81.84

Neryl formate	24.68±1.64	0	0	0	0	0	0	0	0	0	0		0	0	24.76±0.77	0
(E)-linalool oxide (pyranoid)	1125.7±75	2236.27±16.52	2252.24±43.52	2814.64±88.62	206.07±15.66	2761.34±24.29	1362.76±1058.98	849.51±736.78	1021.42±56.5	870.45±764.99	28.83±1.27	1367.65±83.38	587.45±608.3	1531.63±2151.67	87.49±2.73	3919.57±122.44
Citronellol	0	544.56±4.02	0	0	0	268.56±2.36	0	253.69±4.38	297.25±16.44	128.73±3.02	27.42±1.21	0	0	0	0	333.7±10.42
Nerol	21.94±1.46	891.76±6.59	642.97±12.42	359.94±11.33	354.57±26.95	833.13±7.33	268.57±23.12	433.04±7.47	483.19±26.73	341.87±19.93	320.65±18.91	485.74±29.61	147.32±195.57	285.52±137.56	0	1751.51±54.72
Geraniol	4984.91±332.11	6019.29±44.46	7727.35±149.31	3730.83±117.47	1793.29±136.32	7012.65±61.68	1716.29±147.77	3758.05±68.7	4422.31±244.61	3603.71±84.56	2777.29±123.91	6917.19±421.71	3704.28±119.13	1961.36±604.18	0	13064.13±408.11
Benzyl alcohol	821.77±54.75	533.15±3.94	1264.13±24.43	755.23±23.78	0	1068.3±9.4	584.5±50.32	359.22±6.2	481.65±26.64	766.65±116.4	66.99±2.96	787.28±48	316.92±10.19	503.66±256.47	287.59±8.98	1816.8±56.76
Phenylethyl Alcohol	1182.87±78.81	1409.22±10.41	2622.89±50.68	0	978.24±74.36	1614.72±14.2	1099.06±94.63	410.73±7.09	542.02±29.98	1135.89±26.65	196.48±8.68	65.84±4.01	559.62±18	878.75±119.88	1617.45±50.53	3535.25±110.44
Nerolidol	0	0	0	0	0	0	0	0	330.93±18.3	221.62±5.2	0	0	0	0	0	0
T-muurolol	0	0	0	0	0	0	0	0	15.42±10.61	22.23±20.14	0	0	0	0	0	0
**Ketones**																
1-Penten-3-one	105.54±4.24	0	0	85.54±2.69	76.94±11.62	0	20.15±1.74	93.93±9.61	0	0	0	0	0	19.48±3.44	0	100.04±2.21
3-Penten-2-one	0	0	0	0	0	0	0	0	0	62.25±3.93	0	0	0	0	0	0
2,5-dimethyl-3-Hexanone	0	0	0	0	0	121.04±1.06	0	0	0	0	0	0	0	0	167.99±5.25	0
3-Octanone	0	0	0	0	0	0	0	0	0	44.9±1.05	0	0	0	0	0	0
Cistus cyclohexanone	72.28±4.82	27.76±0.21	37.89±0.73	44.06±1.39	45.15±3.43	37.84±0.33	39.5±3.4	35.27±0.61	36.59±2.02	109.1±2.56	10.36±0.46	42.42±2.59	0	18.09±2.71	41.79±1.31	325.81±10.18
6-methyl-5-hepten-2-one	309.03±20.59	99.05±0.73	182.08±3.52	173.1±5.45	150.02±11.4	0	97.37±8.38	203.22±3.51	171.55±9.49	327.83±0.16	48.13±2.13	180.27±10.99	45.66±1.47	51.7±7.76	234.48±7.32	704.09±22
2-Nonanone	0	0	0	0	0	0	0	0	0	149.53±0.4	0	0	0	0	0	291.84±9.12
3-octen-2-one	0	0	105.5±2.04	105.75±3.33	136.88±10.41	0	0	0	106.46±5.89	240.94±5.65	0	44.2±2.69	0	0	190.31±5.95	0
(E)-3-octen-2-one	89.3±5.95	30.35±0.22	0	0	0	33.12±0.29	40.71±3.5	82.7±1.43	0	0	0	0	25.21±0.81	0	0	501.12±15.65
5-Decanone	0	0	0	0	16.14±1.23	0	0	0	0	0	0	0	0	0	0	0
2-Decanone	16.65±1.11	0	0	0	12.65±0.96	0	0	0	0	172.46±4.05	0	0	0	0	0	0
3,5-Octadien-2-one	452.63±30.16	0	213.63±4.13	254.52±8.01	269.41±20.48	0	133.49±18.28	0	727.09±319.06	581.83±168	0	0	181.73±62.54	14.61±2.19	23.41±0.73	0
2-Undecanone	18.04±1.2	0	15.88±0.31	0	0	0	0	0	17.46±0.97	29.01±0.68	0	0	0	0	0	0
Acetophenone	0	28.29±0.21	0	0	0	0	0	22.51±24.28	0	0	12.1±0.53	22.74±1.39	24.8±0.8	0	83.39±2.61	201.83±6.31

Gamma-hexalactone	15.09±1.01	35.06±0.26	0	0	0	0	0	16.33±0.28	16.17±0.89	0	0	15.36±0.94	0	0	29.9±0.93	71.37±2.23
Tetrahydrogeranyl acetone	27.62±1.84	30.1±0.22	33.32±0.64	0	28.9±2.2	0	0	26.88±0.46	17.32±0.96	41.84±0.98	0	27.18±1.66	0	0	0	118.9±3.71
(E)-beta-damascenone	0	79.47±0.59	121.5±2.35	0	0	0	0	22.9±0.4	30.32±1.68	74.58±1.75	0	0	10.72±0.34	40.98±6.15	0	95.19±2.97
α-Ionone	202.11±13.47	0	130.93±2.53	213.33±6.72	0	166.31±1.46	0	157.1±2.71	0	0	9.32±0.41	144.61±8.82	67.68±2.18	0	16559.2±517.3	934.58±29.2
trans-β-Ionone	756.8±50.42	126.41±0.93	310.69±6	0	573.99±43.63	763.87±6.72	0	427.17±7.37	0	580.66±13.63	31.53±1.39	56.29±3.43	237.08±7.62	181.14±27.17	0	4311.92±134.7
**Acids**																
Acetic acid	0	0	59.51±1.15	38.15±1.2	48.94±3.72	0	0	0	186.81±10.33	106.74±2.2	0	0	0	0	0	279.8±8.74
Propanoic acid	0	0	0	0	0	0	0	16.77±0.29	22.06±1.22	0	0	0	0	0	0	0
Isovaleric acid	21.05±1.4	38.57±0.28	0	0	26.79±2.04	0	50.76±4.37	17.97±0.31	0	0	0	0	0	14.89±2.23	32.89±1.03	63.66±1.99
Hexanoic acid	578.37±38.53	92.82±0.69	235.36±4.55	0	612.48±46.56	177.77±1.56	139.65±12.02	0	0	0	0	430.75±26.26	241.3±7.76	150.72±56.29	127.67±3.99	1848.6±57.75
Pentanoic acid	0	0	0	0	16.79±1.28	0	0	0	0	401.45±9.42	0	0	0	0	0	0
**Esters**																
ethyl hexanoate	0	0	0	0	0	0	0	0	0	264.27±6.2	0	387.18±23.6	0	0	0	0
methyl 2-hexenoate	0	0	0	0	0	0	0	0	0	45.92±1.08	0	36.77±2.24	0	0	0	0
methyl (E)-2-hexenoate	65.68±4.38	31.85±0.24	15.82±0.31	16.69±0.53	28.13±2.14	0	0	27.63±0.48	0	0	0	111.65±6.81	21.74±0.7	0	0	64.46±2.01
E-3-hexenyl acetate	0	0	10.72±0.21	0	0	0	0	47.05±0.81	0	0	0	0	45.37±1.46	0	0	0
ethyl 2-hexenoate	0	0	0	45.26±1.43	0	43.75±0.38	0	0	37.71±2.09	87.21±2.05	0	118.09±7.2	0	0	0	0
Hexyl 2-methylbutanoate	0	97.98±0.72	0	0	43.04±3.27	0	31.14±2.68	0	0	0	0	0	0	0	0	109.83±3.43
Ethyl octanoate	0	0	0	0	0	0	0	0	86.57±4.79	74.55±1.75	0	153.12±9.33	0	0	0	0
cis-3-Hexenyl isovalerate	0	92.58±0.68	0	0	83.42±6.34	0	19.43±15.82	66.37±1.15	91.42±5.06	0	21.12±2.15	0	0	11.82±1.77	41.83±1.31	308.7±9.64
Methyl nonanoate	0	0	0	0	0	0	0	0	0	26.25±0.62	0	16.3±0.99	0	0	0	0
Benzoic acid, methyl ester	0	18.53±0.14	0	24.74±0.78	30.31±2.3	0	0	0	0	0	0	0	16.02±0.52	0	0	0
Hexanoic acid, hexyl ester	0	46.64±0.34	0	5.8±0.18	19.94±1.52	0	0	0	0	0	0	0	0	0	0	0
(E)-2-Hexenyl hexanoate	0	0	0	0	13.27±1.01	0	0	0	0	0	0	0	0	0	198.01±6.19	0
Geranyl formate	0	23.96±0.18	68.78±1.33	0	0	0	0	0	0	0	0	0	0	0	0	65.73±2.05

Neryl acetate	0	19.99±0.15	39.39±0.76	0	0	0	0	0	0	0	0	0	0	0	4654.88±145.42	0
Methyl salicylate	8945.68±595.98	12015.52±88.76	10955.61±211.69	10453.49±329.14	9763.82±742.21	4631.57±40.73	3433.43±281.55	3837.4±5390.05	4660.79±6585.99	7374.35±117.6	695.69±39.11	9332.22±568.94	9767.97±261.9	3253.84±1457.79	719.15±22.47	20282.42±633.61
Hexadecanoic acid, ethyl ester	0	0	0	0	0	0	0	0	29.58±1.64	54.17±1.27	0	68.99±4.21	0	0	23.51±0.73	0
**Alkenes and aromatic hydrocarbons**																
Toluene	0	67.64±4.15	99.28±1.64	0	0	0	0	0	96.39±5.33	118.42±7.48	87.03±2.96	0	35.14±1.21	0	155.76±3.44	325.04±7.18
Camphene	0	9.37±0.57	0	0	0	0	0	0	0	0	12.92±0.44	0	0	0	0	117.3±2.59
Ethylbenzene	0	0	0	0	0	0	59±5.08	0	0	18.53±21.93	0	0	0	0	0	104.06±2.3
p-Xylene	27.63±1.11	25.52±1.57	22.4±0.37	0	0	0	0	70.14±7.18	0	0	17.36±0.59	27.69±0.93	0	0	0	760.63±23.76
β-Myrcene	998.72±40.09	749.37±45.97	951.17±15.72	905.37±28.51	410.96±62.05	791.18±6.96	427.07±36.77	1054.18±107.9	980.85±54.25	584.92±225.19	1308.62±106.54	877.54±29.58	429.77±172.91	339.4±12.67	1257.33±27.78	2455.86±76.72
α-Phellandrene	36.1±1.45	58.01±3.56	67.58±1.12	0	0	0	19.8±1.7	0	61.33±3.39	42.68±7.31	0	0	36.48±0.16	0	50.35±1.57	262.11±8.19
Limonene	598.43±39.87	1029.7±7.61	1039.19±20.08	0	288.4±21.92	397.92±3.5	0	794.63±13.71	0	0	40.91±56.32	878.28±53.54	60.16±1.93	0	0	1308.19±40.87
α-Terpinene	72.88±4.86	0	0	0	0	0	0	0	0	0	188.47±8.33	120.49±4.06	0	0	0	100.41±3.14
D-Limonene	0	0	0	0	0	0	0	0	0	747.79±4.9	0	0	0	223.38±33.51	0	0
β-Phellandrene	66.94±4.46	155.49±1.15	23.87±0.46	0	0	0	23.9±2.06	73.03±28.03	115.33±6.38	0	0	0	0	0	0	0
trans-β-Ocimene	269.07±17.93	271.23±2	765.1±14.78	0	0	132.64±1.17	0	0	324.24±17.93	183.04±19.9	730.97±444.45	0	0	76.63±11.5	37.14±1.16	498.22±15.56
γ-Terpinene	56.18±3.74	339.37±2.51	97.55±1.88	0	25.54±1.94	22.52±0.2	0	0	94.04±5.2	73.07±1.71	152±6.72	94.7±5.77	48.75±1.57	0	62.46±1.95	85.11±2.66
Styrene	0	14.11±0.1	15.3±0.3	0	0	181.75±1.6	0	27.58±0.48	33.93±1.88	27.27±0.64	11.24±1.32	0	10.2±0.33	0	0	317.84±9.93
1,3,7-Octatriene, 3,7-dimethyl-	0	0	0	397.88±12.53	0	0	0	343.96±5.94	0	0	0	324.07±19.76	0	0	0	768.88±24.02
o-Cymene	144.76±9.64	0	193.09±3.73	232.96±7.33	45.93±3.49	136.61±1.2	79.89±6.88	230.95±3.99	279.95±15.48	179.86±4.22	231.09±37.08	201.24±12.27	88.68±2.85	47.39±7.11	162.97±5.09	253.22±7.91
Terpinolene	0	0	0	0	0	0	0	0	182.67±10.1	70.24±1.65	0	115.81±7.06	0	0	0	0
(E,E)-2,6-alloocimene	0	83.34±0.62	17.12±0.33	58.25±1.83	0	0	0	57.97±1	47.22±47.74	0	53.77±2.38	0	0	0	0	0
p,α-Dimethylstyrene	0	65.71±0.49	0	74.19±2.34	31.63±2.4	33.68±0.3	0	0	0	61.17±1.44	53.23±2.35	59.34±3.62	0	0	0	130.7±4.08
Theaspirane	308.44±20.55	84.14±0.62	75.33±1.46	80.39±2.53	72.01±5.47	0	67.22±30.39	82.89±6.86	0	139.87±78.57	16.09±0.71	172.16±10.5	39.42±4.94	0	0	359.25±11.22

Estragole	0	30.03±0.22	0	0	0	0	0	0	15.5±0.86	35.81±0.84	0	0	0	0	25.61±0.8	0
